# Understanding the mechanisms of treatment response in depression, focus on electro-convulsive therapy

**DOI:** 10.1007/s00406-020-01184-1

**Published:** 2020-08-31

**Authors:** Elisabeth B. Binder

**Affiliations:** Dept. Of Translational Research in Psychiatry, Max-Planck Institute of Psychiatry, Kraepelinstr. 2-10, 80804 Munich, Germany

Major depressive disorders are among the most common psychiatric disorders, with a lifetime prevalence of up to 20% [1]. While there are a number of effective treatment approaches, including pharmacological, psychotherapeutic or treatments based on invasive or non-invasive brain stimulation, rates of chronic depression and treatment-resistant forms are high. These chronic disease trajectories are not only an enormous burden for the patients and their families, but also for societies, accounting for among the highest years lived with disability [2]. Currently, in the absence of biology-based diagnoses and clinically relevant biomarkers, treatment choices have to remain trial and error, further increasing the time to response for patients. Biomarkers predicting likely response to treatment overall or a specific treatment would be a clinically important advance for psychiatry and are thus a topic of intense research.

The largest number of studies to date have explored biomarkers for predicting or tracking response to antidepressant medication, either overall or to specific classes of antidepressant drugs, with genetic, neuroimaging, or peripheral blood-based biomarkers being the most commonly investigated. Overall, no predictors actually used in clinical routine have yet emerged, including pharmacogenetic markers. Even for genotypes of genes involved in the pharmacokinetics of antidepressant drug, such as the cytochrome P450 gene family, data have remained controversial [3].

Beyond pharmacotherapy, biomarker studies have been scarce, especially for differential response to different treatment modalities, although such biomarkers would be of great value, possibly also allowing to identify biological subtypes of depression. For instance, functional magnetic resonance imaging (fMRI) resting-state functional connectivity analyses with a bilateral subcallosal cingulate cortex seed could predict treatment remission or failure to cognitive behavioral therapy vs. antidepressant drug treatment with over 70% accuracy. Patients with higher summed connectivity with this brain region preferentially remitted to behavioral therapy and did not response to pharmacological treatment. Such differential predictors would not only be on high clinical relevance, but could also shed light on different brain circuit dysfunction in depression relevant for treatment response [4].

In addition to understanding differential treatment response, focusing on the factors underlying treatment response to electroconvulsive therapy (ECT) could also reveal important insight into mechanisms of depression treatment response. ECT is among the most effective forms of treatment for severe depressive disorders, however, it is commonly only applied once other treatment modalities have failed and current guidelines place it at the end of treatment algorithms [5]. This is likely because ECT has more extended logistical demands than other treatments including the use of general anaesthesia and muscle relaxants and associated medical risks and side-effects such as short-term memory impairment. However, given the high response rates to this treatment, even among treatment resistant forms, understanding the biological mechanisms underlying ECT response could help to better understand the biology of major depression and treatment response. Identifying biomarkers to select those patients who will best respond to ECT could help clinicians to implement ECT earlier in the disease course. The topic of biomarkers in ECT is also the focus of two publications in this issue of the European Archives of Psychiatry and Clinical Neuroscience, namely Kranaster et al. and Soda et al. [6, 7]. The two papers in this issue address these specific questions, i.e., identification biomarkers for ECT response but also whether such biomarkers can contribute to our understanding of the pathomechanisms of depression.

Following up on a previous publication exploring how cerebrospinal fluid (CSF) biomarkers correlate with symptom improvement in ECT [8], Kranaster et al., [6] here address the important issue how parameters used to monitor the quality of ECT (similar in principle to therapeutic drug monitoring for pharmacotherapy) correlate with such biomarkers. Specifically, they evaluate how a validated seizure quality index (SQI), based on the extent of several ictal parameters of the seizure that can be assessed at bedside and predicts treatment outcome [9], correlates with the same CSF biomarkers. These include measures of the innate immune system, Tau-protein and beta-amyloids, neurogranin, vascular endothelial growth factor, endocannabinoids, phospholipids and soluble Klotho, covering possible mechanistic factors underlying response to ECT. The authors could show that change in the levels of CSF markers for the innate immune system, for neurodegeneration and from lipids from baseline to after treatment are associated with the SQI. The correlations of these CSF biomarkers with SQI suggest that a proinflammatory profile, higher levels of axonal dysfunction/degeneration are associated with reduced seizure quality and in extent an increased risk for a less-favorable outcome of ECT. Such combination of empirical factors associating with treatment response could shed light into the mechanisms involved in response to ECT and to preselect patients with a likely favorable response.

Soda et al. [7] present another approach for understanding factors contributing to ECT response, namely genetic association studies. Like any other complex trait, response to ECT will be determined by polygenic factors, each with small effects, necessitating large sample sizes to detect meaningful and robust associations. Large scale, international consortia, such as for example the Psychiatric Genomics Consortium, have been instrumental in advancing our knowledge on the genetic underpinnings of psychiatric disease [10]. This manuscript here presents the International Consortium on the Genetics of Electroconvulsive Therapy and Severe Depressive Disorders in short, Gen‑ECT‑ic, that has the goal to assess genome-wide genetic associations with response to ECT in a sample of about 30,000 patients with severe major depression. In addition to investigating the genomic underpinnings of severe, treatment-resistant depression and the genetic contribution to treatment response to ECT, this consortium will also investigate genetic factors associated with the cognitive side effects of ECT. If successful, results from these analyses could point to biological pathways relevant for severe depression as well as response to ECT and its side effects.

Such polygenic markers, in combination with clinical information as well as other biomarkers, including neuroimaging and not only blood- but also CSF-based biomarkers could optimize matching of patients to the treatment they will most likely respond to with the least side effects. Optimally, such personalized approaches will reduce the rates of chronic and treatment resistant depression and improve the quality of life of many patients with depression.
